# Description and Clinical Implications of Myocardial Clefts Using Echocardiography

**DOI:** 10.7759/cureus.15407

**Published:** 2021-06-02

**Authors:** Ghaith Alhatemi, Aditya Sood, Haider Aldiwani, Rafal Alhatemi, Abdelrahman Ahmed, Mohamed Shokr, Mohamed Zghouzi, M. Chadi Alraies, Shaun Cardozo

**Affiliations:** 1 Internal Medicine, Wayne State University, Detroit, USA; 2 Cardiology, Conway Medical Center, Conway, USA; 3 Internal Medicine, Scripps Mercy Hospital San Diego, San Diego, USA; 4 Internal Medicine, Wayne State University, Detroit Medical Center, Detroit, USA; 5 Pulmonary and Critical Care Medicine, Mayo Clinic, Rochester, USA; 6 Cardiology, New York University Grossman School of Medicine, New York, USA; 7 Internal Medicine, Detroit Medical Center, Detroit, USA; 8 Cardiology, Detroit Medical Center, Detroit, USA; 9 Cardiology, Wayne State University, Detroit, USA

**Keywords:** myocardial clefts, myocardial crypts, hypertrophic cardiomyopathy, 2-dimensional echocardiography, cardiac magnetic resonance imaging

## Abstract

Background

Myocardial clefts (MCs) are rare anomalies with debatable clinical significance. Increased use of cardiac magnetic resonance (CMR) has led to the appreciation of subtle left ventricular (LV) wall structural defects, and studies showed varying clinical significance, ranging from asymptomatic incidental findings to being considered a novel imaging marker of hypertrophic cardiomyopathy. Sparse data are available about the utility of two-dimensional echocardiography (2DE) to visualize these anomalies. We describe our institutional experience categorizing MCs using 2DE.

Methods

The echocardiography database was retrospectively queried for diagnosing MCs using Synapse® Cardiovascular Picture Archiving and Communication System (PACS) (Fujifilm, Tokyo, Japan). Identified patients were admitted to Detroit Medical Center (DMC) between January 2012 and May 2019. MCs were defined as recesses filled with luminal blood, obliterate during systole, and have U, wedge, and tunnel shapes. Images were interpreted by a cardiologist blinded to the data. Baseline demographics and clinical characteristics were documented. The study was descriptive; no intervention was done.

Results

Sixteen patients with a mean age of 62.43 were included; 68.75% were women, and 81.25% were African American. The prevalence of cardiac comorbidities was primary hypertension 12 (75%), coronary artery disease 5 (31.25%), heart failure with reduced ejection fraction (HFrEF) 4 (25.0%), valvular heart disease 4 (25.0%), arrhythmia/heart block 4 (25.0%), and heart failure with preserved ejection fraction (HFpEF) 2 (12.5%). The indications for 2DE evaluation were heart failure/cardiogenic shock 2 (12.5%), acute coronary syndrome 2 (12.5%), syncope/presyncope 2 (12.5%), atypical chest pain 2 (12.5%), and others 8 (50.0%). Twenty-one MCs were visualized in eight segments of LV walls and septum as follows: basal inferior 7, mid inferoseptal 6, mid inferior 3, mid anteroseptal 2, mid inferolateral 1, mid anterolateral 1, basal inferoseptal 1, apical inferoseptal 1, and apical septal 1. Morphology was classified as tunnel in 66.66%, wedge in 23.8%, and U in 9.5%.

Conclusion

In various LV and septal walls, MCs detected on 2DE were benign and incidental findings without significant implications for preclinical hypertrophic cardiomyopathy (HCM).

## Introduction

Myocardial clefts (MCs) are narrow, deep invaginations within the myocardium, localized predominantly in the basal inferior septum and left ventricular (LV) free walls [1]. The terms "crypts" and "clefts" have been used interchangeably to describe recesses containing luminal blood into the otherwise normal compact myocardium [1]. There is no consensus on an exact definition of the clefts or crypts, although previous studies described them on advanced imaging as “a discrete V- or U-shaped extension of blood that penetrates more than 50% of the adjoining myocardial thickness in diastole and exhibits near-complete obliteration in systole” [2,3]. Partial crypts were also defined as recesses penetrating 25%-50% of the wall thickness [3]. A classification proposed by some authors suggested the term “recess” identifies these “partial crypts” [1].

Echocardiography was used earlier for imaging of myocardial structural defects. However, its accuracy can be challenging, particularly if the defect's location does not coincide with standard acquisition planes [1,4]. 

Although clefts or crypts may be significant findings in the context of a high pretest probability of HCM [5,6], their clinical significance is questionable when detected as isolated findings in other individuals, probably representing mere incidental and benign variants of myocardial structure [2,3]. We present our institutional experience with MCs using echocardiography.

## Materials and methods

Patient selection and baseline characteristics

Using the Synapse® Cardiovascular Picture Arching and Communication System (PACS) (Fujifilm Medical, Tokyo, Japan) search option, our echocardiography database was retrospectively queried by the following keywords: clefts, crypts, fissures, or crevices. Seventeen patients were identified. For this report, myocardial clefts (MCs) were used synonymously with crypts, fissures, and cervices. Patients included in the analysis were admitted to Detroit Medical Center/Wayne State University (Detroit, MI, USA) between January 2012 and May 2019 and had two-dimensional echocardiography (2DE) for various indications (Table 1). This study was approved by the Institutional Board Review (IRB). Baseline demographic, electrocardiographic, and clinical characteristics were documented. In one subject, the formal echocardiography report was interpreted as a “cleft”; after a careful review of the echocardiography images and cardiac coronary computed tomography (cCTA) conducted for a different indication, the diagnosis was changed to a diverticulum, and that patient was excluded from the analysis. In another subject (#16), a cCTA confirmed the diagnosis of two MCs with their locations corresponding to the echocardiogram findings. No cardiac computed tomography or cardiac magnetic resonance (CMR) was done in the remainder of the population.

**Table 1:** Baseline characteristics and demographics of patients with myocardial cleft

Baseline characteristics and demographics of patients with myocardial cleft	
Age, Mean (SD)	62.43 (20.23)
Female, No. (%)	11 (68.75)
Race	No. (%)
African American	13 (81.25)
Caucasian	3 (18.75)
Cardiac Comorbidity Prevalence	No. (%)
Primary hypertension	12 (75.00)
Coronary artery disease	5 (31.25)
Heart failure with reduced ejection fraction	4 (25.00)
Valvular heart disease	4 (25.00)
Arrythmia/heart block	4 (25.00)
Heart failure with preserved ejection fraction	2 (12.50)
Indications for Echo Referral	No. (%)
Heart failure/cardiogenic shock	2 (12.5)
Acute coronary syndrome	2 (12.5)
Atypical chest pain	2 (12.5)
Syncope/presyncope	2 (12.5)
Others	8 (50.0)

Transthoracic echocardiograms were obtained using a variety of techniques, including two-dimensional imaging. Myocardial contrast echocardiography with Definity® (Perflutren Lipid Microsphere, Lantheus Medical Imaging, Inc., North Billerica, MA) was done in six subjects (#6, 10, 11, 13, 14, and 16), and biplane echocardiography was performed on three subjects (#14, 15, and 16), which aided in the diagnosis. A variety of views were obtained for resting imaging, including but not limited to the parasternal long-axis, parasternal short-axis (at multiple levels), apical two-chamber, apical three-chamber, and apical five-chamber views.

Image analysis and definition of clefts

All routine 2DE images were processed using commercially available PACS. MCs were defined as structural abnormalities composed of narrow, deep blood-filled invaginations that penetrate the adjoining myocardium during diastole and obliterate during systole. Due to the inconsistency in MCs classification and the lack of good spatial resolution as seen with CMR, we included any depth of myocardial thickness penetration. The overall appearance of the MCs was divided into three groups: triangular (wedge-shaped), a width half the height (tunnel-shaped), or little difference in width and height (U-shaped). Their locations were reported based upon the 17-segment heart model recommended by the American Heart Association. To minimize interobserver variability, the images were reviewed by two experts blinded to the patient’s data.

Further echocardiographic parameters were measured and included interventricular septal thickness in diastole and posterior wall thickness (PWT). Ejection fraction (EF) was assessed visually. Geometrical assessment of LV was conducted by calculating LV relative wall thickness (RWT) and LV mass. A formula (2 × PWT)/(LV internal diameter at end-diastole) was used for RWT calculation, and a cutoff of 0.42 mm was used for the upper limit of normal for both genders. Using the linear method, LV mass was then calculated by Cube formula, and the following reference values were utilized (67-162 g for women and 88-224 g for men) [7]. LV mass was not indexed to body surface area. Data were presented as mean ± standard deviation.

## Results

Patients' characteristics

Sixteen patients with a mean age of 62.4 (±20.2) were included; 68.8% were women, and 81.3% were African American. Prevalence of cardiovascular comorbidity was primary hypertension 12 (75.0%), coronary artery disease 5 (31.25%), heart failure with reduced ejection fraction (HFrEF) 4 (25.0%), valvular heart disease 4 (25.00%), arrhythmia/heart block 4 (25.0%), and heart failure with preserved ejection fraction (HFpEF) 2 (12.5%). Indications for evaluation whether outpatient visit or in-hospital admission were heart failure/cardiogenic shock 2 (12.5%), acute coronary syndrome 2 (12.5%), syncope/presyncope 2 (12.5%), atypical chest pain 2 (12.5%), and others 8 (50.0%). Table 1 summarizes the baseline demographic and clinical criteria for the patients with MCs.

Myocardial clefts characteristics

**Figure 1:**
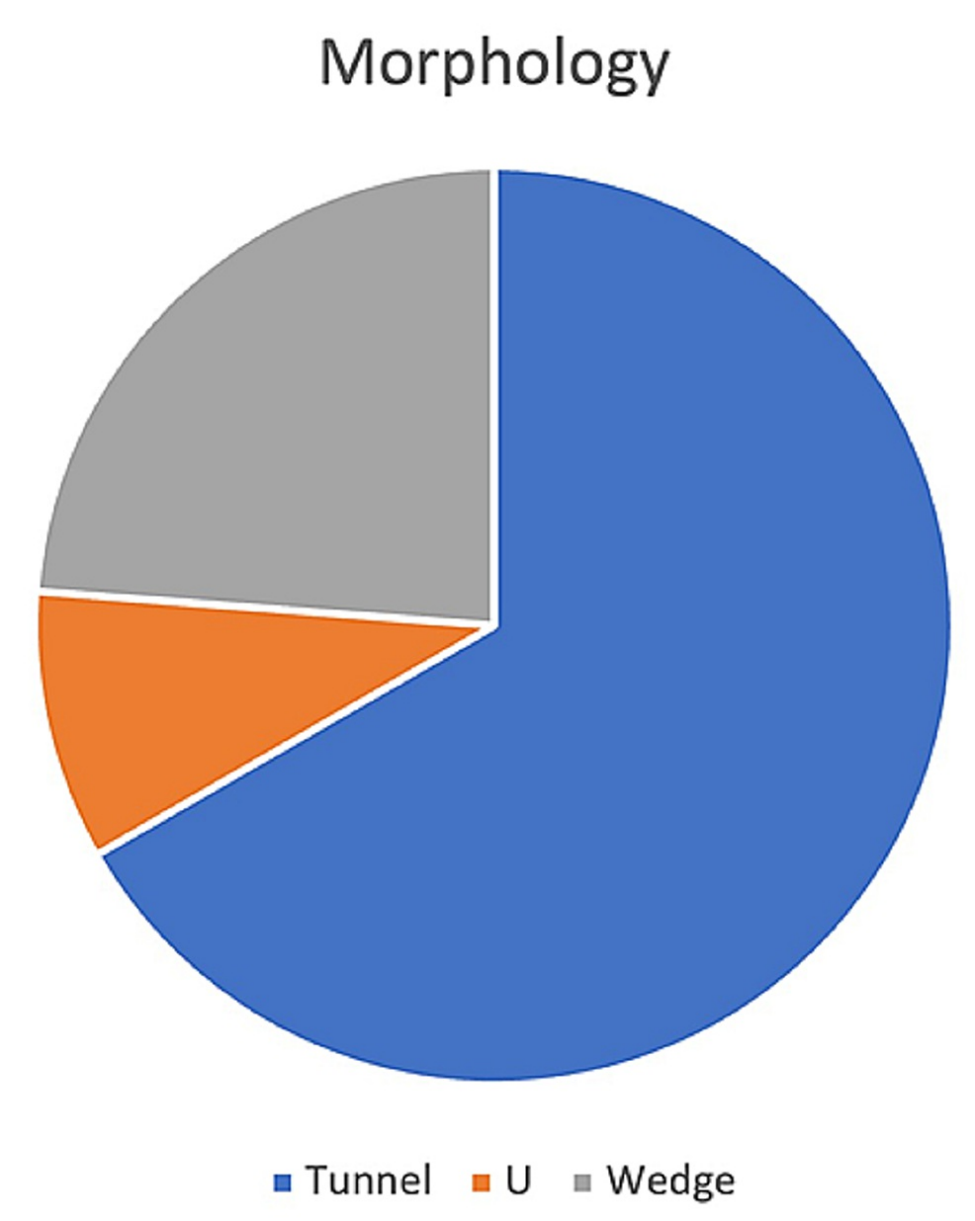
Distribution of morphology Most clefts (14) were tunnel-shaped (66.6%), whereas five (23.8%) were wedge- and two (9.5%) were U-shaped.

**Figure 2:**
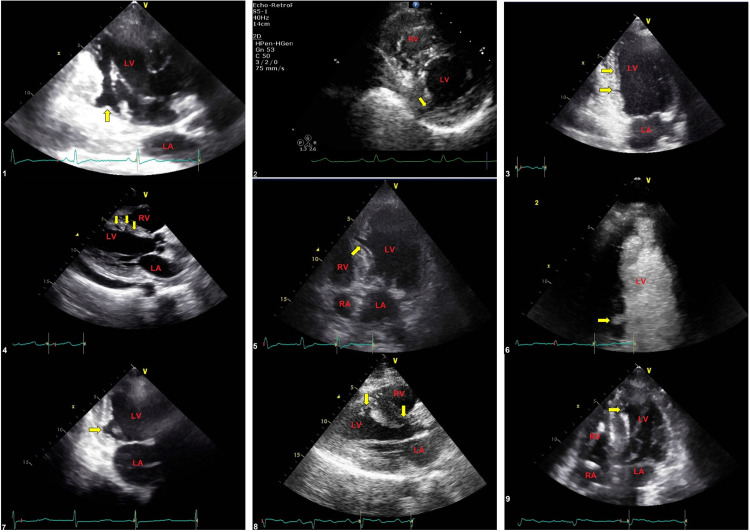
Image illustrates multiple long-axis and short-axis views of echocardiography showing different types of clefts (yellow arrows) and cardiac chambers (labeled in red) The images are numbered by the patients from 1 to 9. For more information about these clefts, please correlate with Table 3 by patient sequence.

**Figure 3:**
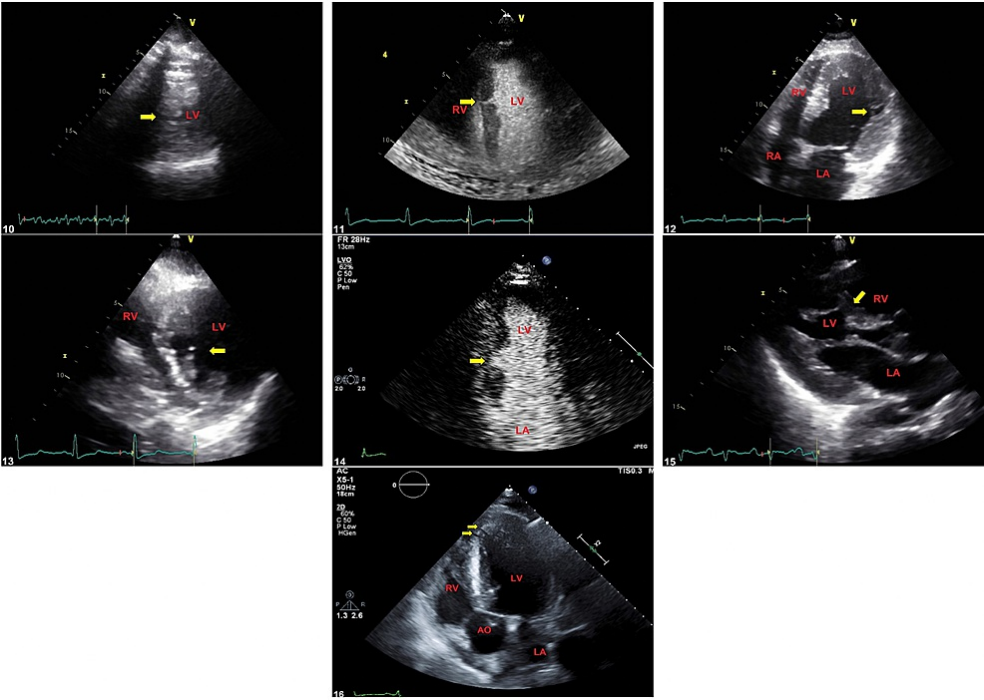
Image illustrates multiple long-axis and short-axis views of echocardiography showing different types of clefts (yellow arrows) and cardiac chambers (labeled in red) The images are numbered by the patients from 10 to 16. For more information about these clefts, please correlate with Table 3 by patient sequence.

A total of 21 MCs were identified in all patients, with a minimum of one and a maximum of three. MCs visualized in eight American Heart Association (AHA) myocardial segments all confined to the LV wall and septum as follows: basal inferior 7, mid inferoseptal 6, mid inferior 3, mid anteroseptal 2, mid inferolateral 1, mid anterolateral 1, basal inferoseptal 1, apical inferoseptal 1, and apical septal 1. Morphology was classified as tunnel shape in 66.7%, wedge shape in 23.8%, while 9.5% had U shape. Table 2 summarizes these clefts' locations. Figure 1 illustrates the distribution of morphology, while Figures 2, 3 show the entire clefts as imaged by 2DE.

**Table 2:** Echocardiographic locations of myocardial clefts using the AHA seven-segment heart circumferential polar plot This table illustrates the locations of the clefts imaged on 2DE, with the numbers corresponding to the count of clefts visualized in each segment; note that the same cleft might be visualized in more than one segment, and thus the segment count might outnumber the clefts. AHA, American Heart Association; 2DE, two-dimensional echocardiography.

Basal Segments		Mid Cavitary Segments		Apical Segments	
Location	No.	Location	No.	Location	No.
1. Basal anterior	0	7. Mid anterior	0	13. Apical anterior	0
2. Basal anteroseptal	0	8. Mid anteroseptal	2	14. Apical septal	1
3. Basal inferoseptal	1	9. Mid inferoseptal	6	15. Apical inferior	0
4. Basal inferior	7	10. Mid inferior	3	16. Apical lateral	0
5. Basal inferolateral	0	11. Mid inferolateral	1	17. Apex	0
6. Basal anterolateral	0	12. Mid anterolateral	1		

**Figure 4:**
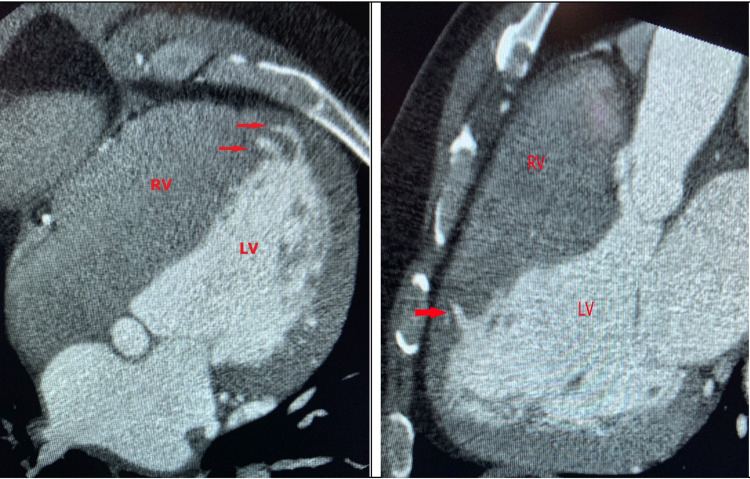
Patient #16’s CTA images of the clefts (red arrows) and ventricular structures (labeled in red); the clefts are extending from the anterior septum to the inferior septum CTA, Coronary computed tomography.

Using the reference values for LV geometry measurements from the American Society of Echocardiography and the European Association of Cardiovascular Imaging [7], concentric remodeling was detected in five subjects, while concentric LV hypertrophy was seen in eight subjects. Three individuals had normal LV geometry. The hypertrophy and remodeling were explained in most subjects by primary hypertension, valvular heart disease, HFrEF, HFpEF, either alone or in combination. In subject #16, despite primary hypertension, there was a pattern of increased apical septal thickness with obliteration in the apical lumen as evaluated by 2DE. cCTA, however, showed that maximum LV wall thicknesses in diastole were 11 mm in the septal segment, a finding that goes against HCM. The two MCs were seen in the apical septal wall extending from the anterior septum to the inferior septum. Figure 4 illustrates the clefts on cCTA. Table 3 provides a more comprehensive review of the clinical, electrocardiographic, and echocardiographic characteristics for the individual subjects.

**Table 3:** Baseline demographic, clinical, electrocardiographic, and echocardiographic criteria of individual subjects with myocardial clefts CV = Cardiovascular; L x D = length x depth; M = morphology; L = location; EF = ejection fraction; IVDST = interventricular septal diastolic thickness; LVDD = left ventricular diastolic diameter; LVPWT = left ventricular posterior wall thickness; RWT = relative wall thickness; LVM = left ventricular mass; HCM = hypertrophic cardiomyopathy; LVG = left ventricular geometry; T = tunnel; W = wedge; I = inferior; IS = inferoseptal; IL = inferolateral; AS = anteroseptal; AL = anterolateral; HFpEF = heart failure with preserved ejection fraction; HFrEF = heart failure with reduced ejection fraction; AR = aortic regurgitation; TR = tricuspid regurgitation; MS = mitral stenosis; SSS = sick sinus syndrome; RBBB = right bundle branch block; pAfib = paroxysmal atrial fibrillation; cAFib = chronic atrial fibrillation; PVCs = premature ventricular contractions; CAD = coronary artery disease; LVH = left ventricular hypertrophy; HTN = primary hypertension; LAD = left atrial dilatation; PRES = posterior reversible leukoencephalopathy; cR = concentric remodeling; NG = normal geometry; cLVH = concentric left ventricular hypertrophy; NSTEMI = non-ST-elevation myocardial infarction.

Demographics			Clinical and Electrocardiographic Criteria			Clefts				Echocardiographic Criteria							
Subject	Age	Gender	Evaluation Reason	Cardiac Conditions	EKG	#	L x D	M	L	EF	IVSDT	LVDD	LVPWT	RWT	LVM	HCM	LVG
1	60	M	Syncope	HTN	Left anterior hemi-block	1	1.3 x 0.82	U	Basal I	60%-65%	1.61	4.16	1.034	0.49	205.07	No	cR
2	30	M	Atypical chest pain	HTN	Early repolarization in inferolateral leads, P mitrale	1	0.85 x 1.06	T	Mid I	55%-60%	0.87	5.04	1	0.39	172.51	No	NG
3	18	F	Asthma exacerbation	None	Sinus tachycardia	2	0.65 x 0.61	T	Mid I	80%	0.78	4.1	1.05	0.51	118.95	No	cR
							0.6 x 0.67	T	Basal I								
4	48	F	Acute on chronic HFpEF	AR, HFpEF	P mitrale, small voltage QRS	3	1.1 x 1.48	T	Mid IL	55%-60%	1	4.1	1.1	0.53	143.75	No	cR
							1 x 0.67	T	Mid IS								
							0.85 x 0.95	T	Mid IS								
5	87	F	Heart murmur	MS, HTN	Not done	1	1.8 x 1.2	T	Mid IS	65%-70%	1.6	3.5	1.1	0.62	164.59	No	cLVH
6	82	M	Cardiogenic shock	SSS, CAD	LAD, ST/T wave inversion in anterolateral leads	1	0.82 x 1.01	U	Basal I	10%-15%	1.4	4.1	1.149	0.56	190.02	No	cR
7	77	F	Seizures due to PRES	HTN, CAD, pAfib, TR	PVCs, junctional bradycardia	1	1.9 x 1.064	W	Basal I	55%-60%	1.01	4.4	0.88	0.4	139.5	No	NG
8	49	F	HTN evaluation	HTN, MS	LAD, LVH, T wave inversions in anterolateral leads	2	1.0 x 0.95	W	Mid AS	55%-60%	1.63	4.1	1.76	0.85	295.06	No	cLVH
							0.94 x 1.2	T	Basal I								
9	93	F	Presyncope	HTN, cAfib, HFrEF	Ventricular paced rhythm	1	0.92 x 1	T	Mid IS	60%-65%	1.62	3.25	1.2	0.73	159.21	No	cR
10	80	F	Acute kidney injury	HTN, HFpEF	RBBB	1	1.3 x 0.7	T	Basal IS	55%-60%	1.2	5.1	1.3	0.51	259.71	No	cLVH
11	54	F	Malignant ascites	WNL	Normal sinus rhythm	1	0.65 x 0.51	T	Mid IS	55%-60%	0.76	4.59	0.7	0.3	107.48	No	NG
12	68	M	Preoperative evaluation	HFrEF, CAD, pAfib, HTN	T wave inversion in inferolateral leads	1	1.1 x 1.3	W	Mid AL	20%-25%	2.4	3.1	1.6	1.03	274.55	No	cLVH
13	83	F	NSTEMI type 2	CAD, HTN	New ST & T depressions in anterolateral leads	1	1.3 x 1.1	T	Basal I	55%-60%	2.051	4.2	1	0.47	258.52	No	cLVH
14	60	F	Atypical chest pain	CAD, HTN	Atrial fibrillation	1	2.6 x 0.74	W	Basal-mid I	55%-60%	1.2	5.2	1.1	0.42	239.11	No	cLVH
15	50	F	NSTEMI type 2	HTN	Sinus tachycardia	1	1.2 x 1.023	W	Mid AS	55%-60%	0.95	4.6	1.2	0.52	178.61	No	cLVH
16	60	M	Unspecified troponin elevation	HFrEF, HTN	T wave inversion in anterolateral leads	2	0.75 x 0.78	T	Apical S	55%-60%	1.8	4.5	1.8	0.8	369.85	No	cLVH
							0.73 x 1.1	T	Mid IS								

## Discussion

Cardiovascular imaging

This study addresses the role of 2DE in describing MCs. Contrast echocardiography was employed in six subjects (37.5%), while biplane imaging was conducted in only three subjects (18.75%). The diagnostic yield of these techniques had not been thoroughly evaluated in the literature.

Most of our information about echocardiography is based upon individual case reports [8-10]. These reports described blood-filled invaginations of the myocardium with near-total obliteration in systole. In two reports, contrast-enhanced harmonic imaging allowed further characterization and confirmation of these clefts [8,10]. While echocardiography can sometimes detect these clefts, the sensitivity and specificity of these pathological findings have been questioned [4]. For instance, in two CMR studies, clefts (termed "crypts" in these studies) were seen in a significant proportion of hypertrophic cardiomyopathy (HCM) mutation carriers; however, none of these clefts were detected on 2DE findings that led authors to conclude that 2DE is not reliable in imaging of these clefts and that contrast use with this technique has to be evaluated [5,6].

On the other side, multiplanar CMR has high spatial resolution and increased contrast between blood and endocardial border surface and has led to the appreciation of subtle LV wall structural features, not seen or neglected by 2DE [4]. Most studies that delineated clefts used standardized imaging protocols with four-, three-, and two-chamber vertical longitudinal axis (VLA) cines [5,6,11]. Another CMR study showed that a modified two-chamber cine through the inferoseptal area doubled the sensitivity to detect clefts compared with the standard long-axis views [12]. The majority of clefts in our study were visualized in the basal inferior and mid inferoseptal LV walls, findings reminiscent of other studies [3,5]. The basal inferior LV wall is the region of insertion between the free walls of the LV, RV, and the interventricular septum, a region believed to be subject to myocardial disarray, described in postmortem studies in hearts of some healthy individuals as well as those with HCM [13].

Clinical significance and comparison to other studies

Our study findings remain in keeping with previously published data in healthy volunteers and other cardiac diseases including primary hypertension [2,3,11] and support the concept that MCs may represent benign and incidental findings.

The most common comorbid condition in our cohort of patients was primary hypertension followed by coronary artery disease. An earlier study by Johansson et al. described 27 basal inferior and 24 septal clefts among 399 CMR cases studied retrospectively. In that study, MCs were found in 15.6% of healthy volunteers (13/120), 5.5% of HCM patients (5/19), and 11.4% of hypertensive patients (5/44) [2]. In a more recent retrospective analysis on 686 consecutive patients who had undergone clinically requested CMR, clefts (termed "crypts" in the study) were identified in 6.7% of the study population, with the highest prevalence in HCM (15.6%), myocarditis (15.4%), and hypertension (13.6%) [3]. In another larger size (n = 1020) CMR study with a similar design, MCs were more frequently found in the HCM group (9/76, 12%) and in hypertensive cardiomyopathy (3/11, 27%); MCs, however, were found less frequently in their control group (11/306, 3.6%) [11]. Our overall reported prevalence of MCs in primary hypertension is thus in concordance with these reports; it is worth mentioning that a 50% myocardial thickness involvement was used as a criterion in these studies.

On the other side, two other studies with different designs utilizing CMR suggested the use of MCs as a novel cardiovascular imaging marker for HCM that can potentially identify HCM family members who should be offered genetic testing [5,6]. One of these prospective studies was done by German et al. and had demonstrated clefts (termed crypts) in 81% of HCM mutations carriers (13/16) and none in their control counterparts (0/16) [5]; a similar more recent larger study disclosed a prevalence of 4% (10/261) in HCM patients, 61% (19/31) in mutation carriers, and similarly no clefts were visualized in the control group (0/98) [6]. Both studies are prospective, investigated a predefined cohort of abnormal sarcomeric protein carriers for HCM, and used a control group without LV hypertrophy or family history of HCM. It is interesting that the relatively low prevalence of these clefts in frank HCM in the latter study was theorized to be attributed to the possibility of regression of these invaginations with subsequent LV wall thickening and remodeling [6], a finding that contrasts our observation as a significant proportion of our cohort has echocardiographic evidence of LV hypertrophy and remodeling.

Other myocardial structural anomalies

Myocardial clefts or crypts must be differentiated from diverticula, aneurysms, and pseudoaneurysms. Morphologically and histologically, clefts and muscular diverticula are very similar in that both have all three layers (endocardium, myocardium, and epicardium), have a narrow neck and a thick myocardial wall, and exhibit myocardial contractility during systole; however, diverticula extend beyond the myocardial wall into the epicardium [1]. Therefore, differentiation between these anomalies by the means of 2E only can be challenging.

Study limitations

Our study was retrospective, single-center, and has a small sample size, and thus observations might not be generalizable to a large cohort of the population. In addition, with the lack of sharp contrast between endocardial border and blood with the use of CMR, the cleft definition was not accurately assessed. Left ventricular geometry measurements with 2DE are not as accurate as 3D echo and CMR - both techniques were not used in our study. We suggest a multicenter prospective registry with a large number of patients imaged by both CMR and 2DE to investigate the utility and accuracy of echocardiogram in the diagnosis.

## Conclusions

In conclusion, MCs are generally benign and incidental findings and can be detected in a variety of patients without significant implications for preclinical HCM.
